# Perceptual Grouping Enhances Visual Plasticity

**DOI:** 10.1371/journal.pone.0053683

**Published:** 2013-01-02

**Authors:** Tommaso Mastropasqua, Massimo Turatto

**Affiliations:** 1 Center for Mind/Brain Sciences, University of Trento, Rovereto, Italy; 2 Department of Cognitive Sciences and Education, University of Trento, Rovereto, Italy; University of Gent, Belgium

## Abstract

Visual perceptual learning, a manifestation of neural plasticity, refers to improvements in performance on a visual task achieved by training. Attention is known to play an important role in perceptual learning, given that the observer's discriminative ability improves only for those stimulus feature that are attended. However, the distribution of attention can be severely constrained by perceptual grouping, a process whereby the visual system organizes the initial retinal input into candidate objects. Taken together, these two pieces of evidence suggest the interesting possibility that perceptual grouping might also affect perceptual learning, either directly or via attentional mechanisms. To address this issue, we conducted two experiments. During the training phase, participants attended to the contrast of the task-relevant stimulus (oriented grating), while two similar task-irrelevant stimuli were presented in the adjacent positions. One of the two flanking stimuli was perceptually grouped with the attended stimulus as a consequence of its similar orientation (Experiment 1) or because it was part of the same perceptual object (Experiment 2). A test phase followed the training phase at each location. Compared to the task-irrelevant no-grouping stimulus, orientation discrimination improved at the attended location. Critically, a perceptual learning effect equivalent to the one observed for the attended location also emerged for the task-irrelevant grouping stimulus, indicating that perceptual grouping induced a transfer of learning to the stimulus (or feature) being perceptually grouped with the task-relevant one. Our findings indicate that no voluntary effort to direct attention to the grouping stimulus or feature is necessary to enhance visual plasticity.

## Introduction

One of the more remarkable properties of the human brain is plasticity, the ability of the brain to change through experience [1]. Sensory plasticity, for example, refers to modifications in the functional and neural organization of the cerebral cortex that occur after substantial changes in the sensory input, either through a massive exposure to behaviorally relevant stimuli or through sensory deprivation [2]. Perceptual learning [3], an experience-dependent improvement in discriminative ability that may occur for different sensory inputs [4]–[6], is considered evidence that plasticity is retained in the adult brain [7]–[10] beyond the initial critical period after birth [11].

Numerous studies have addressed perceptual learning in vision and have suggested that the main factors involved in governing this process and in gating visual plasticity are attention and reinforcement signals [12]. Top-down models posit that learning occurs only for the task-relevant feature of the stimulus that is intentionally attended [13], [14], whereas no learning is observed for the unattended features of the same stimulus. However, recent studies on task-irrelevant perceptual learning have challenged this interpretation. Learning may also occur for subliminal unattended stimuli or features when they are paired extensively and repeatedly with reinforcement signals. These signals would be triggered by the rewarding experience associated with the sense of accomplishment that follows the correct identification of supraliminal targets [15]–[16] or released when exogenous primary rewards (e.g., water for thirsty observers) are delivered [17].

Psychophysical studies on visual plasticity have shown that perceptual learning is usually specific regarding the parameters of the sensory input. Learning can remain confined to the trained eye [18], the stimulus spatial frequency [19], retinotopic location [19], [20], direction of motion [21], and orientation [13], [19]. This lack of generalization has been taken as evidence that perceptual learning may reflect changes in early visual areas [22]–[24]. However, more recent research has challenged this view, emphasizing the possible involvement of higher-level cortical areas in perceptual learning processes [25], [26].

In addition to the analysis of the basic features of a given stimulus, the visual system must solve, at different stages of analysis, the important problem of perceptual organization [27], [28]. As first noted by the Gestaltists [29], visual objects, as we consciously perceive them, do not exist in the pattern of retinal stimulation. In the retina, the image of the external world is decomposed into spatially independent single inputs that are registered by photoreceptors and transmitted, via the optic nerve, to the visual cortex. In the visual cortex the different visual inputs must be reconstructed in a meaningful way. This process is known as perceptual grouping [29]. Grouping is obtained by means of a set of “Gestalt principles” – such as for example similarity, proximity, closure – that organize fragments of the retinal image into candidate objects by bundling together those parts that are likely to belong to the same object in the real world [30]. The results of these grouping processes are segmented perceptual units, or “proto objects”. These “pre-attentive” elements form the basis for further in-depth analysis, such as conscious object recognition [31].

The processes involved in perceptual organization have profound effects on the distribution of attention among the different stimuli presented in the visual field. For example, stimuli that form perceptual units with the attended one are also automatically selected by attention [32]. Attention, on the other hand, plays a fundamental role in perceptual learning, gating visual plasticity [13], [14]. This raises the interesting possibility that perceptual grouping might affect perceptual learning, either directly or by means of attention.

To address this issue we adapted a recently proposed paradigm showing that attention alters the degree of visual plasticity during exposure-based perceptual learning [33]. This paradigm appears to be particularly suited to isolate the role of attention on perceptual learning, without the possible contribution of rewarding mechanisms.

## Materials and Methods

Except for few aspects detailed below, we adopted the same paradigm proposed by Gutnisky et al. [33] both in Experiment 1 and Experiment 2.

### Ethic statements

The study was approved by the local institutional ethics committee (Comitato Etico per la Sperimentazione con l'Essere Umano, University of Trento, Italy). Written informed consent to participate in the study was obtained from all participants, and the experiments were carried out in accordance with the Declaration of Helsinki.

### Participants

Thirty-two (24 females; mean age  = 21) paid volunteers participated in Experiment 1, and thirty-eight in Experiment 2 (30 females; mean age  = 21). All had normal or corrected-to-normal vision and were naïve as to the purpose of the experiment.

### Apparatus

Stimuli were presented on a ViewSonic 690 CRT 19′′ monitor (1024×768, 100 Hz). The generation and presentation of the stimuli was controlled by a custom-made program written using Matlab and the Psychophysics Toolbox 3.8, running in Windows 2000 on a Pentium IV Dell PC. Fixation was monitored by means of an eye tracker system (EyeLink 1000 Desktop Mount, SR Research; 500 Hz sampling rate).

### Stimuli

**Figure 1:**
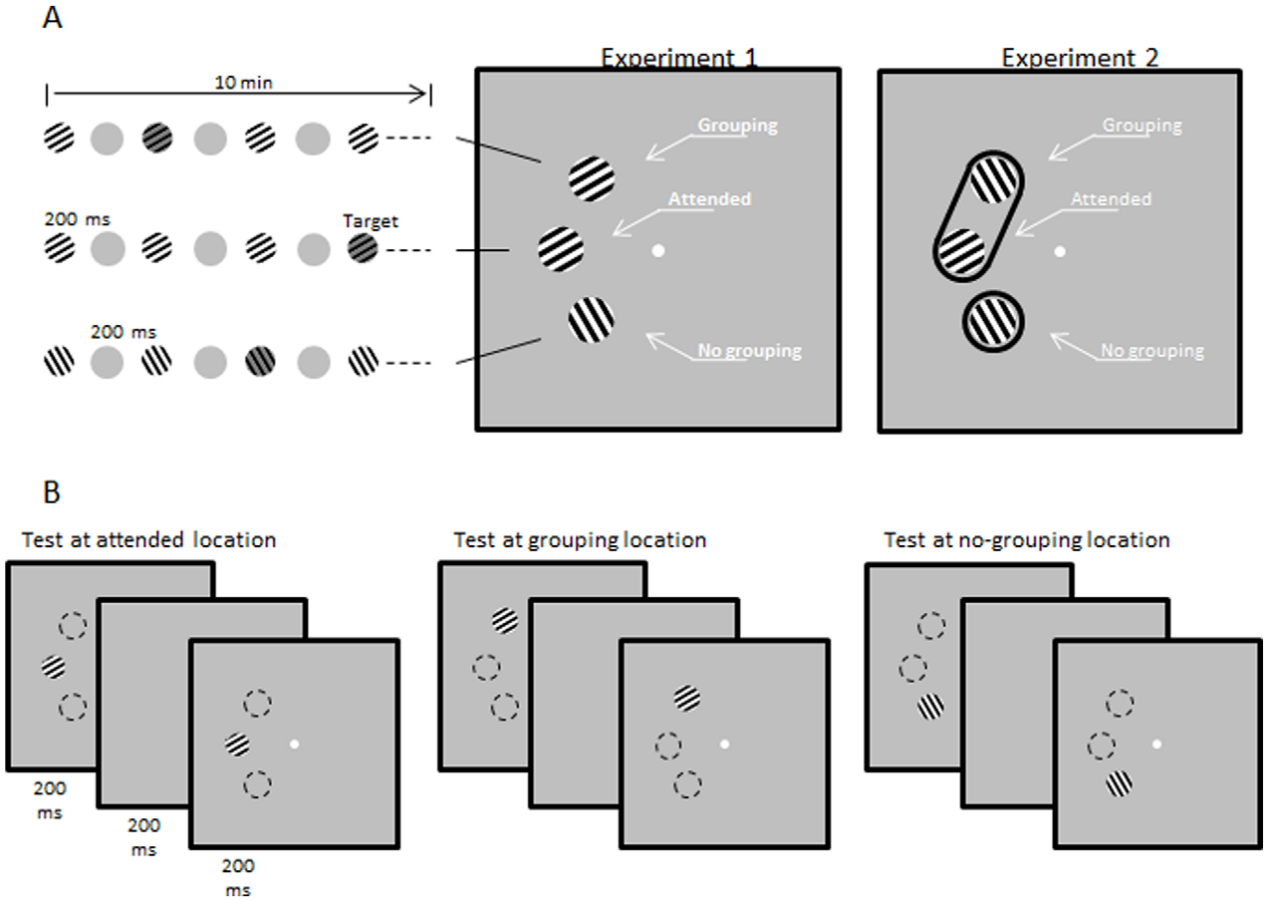
Experimental procedures for training and test phases. (A) Schematic representation of the paradigm used in the exposure phase of Experiments 1 and 2. Three oriented gratings, equidistant from the central fixation point, appeared at three different spatial locations (attended, grouping, no-grouping) in a rapid alternating series. The gratings were simultaneously flashed for 200 ms, and consecutive presentations were separated by 200-ms blank intervals. Participants were presented with 5 such series of alternating gratings, lasting 2 min each (for a total duration of about 10 min). At each location, gratings appeared with a lower contrast in a small proportion (10%) of presentations. The order in which lower-contrast gratings appeared at a given location was randomly assigned. The participants' task was to detect the occurrence of lower-contrast gratings at the attended location, while ignoring the two flanking locations. In Experiment 1, perceptual grouping was based on orientation similarity. In the example depicted here, the upper grating shares the same orientation (60°) as the attended grating (grouping condition), whereas the lower grating, orthogonally tilted (150°), forms a distinct perceptual unit (no-grouping condition). In Experiment 2, grouping was obtained using an ellipse-like black shape. The gratings at the grouping and no-grouping locations were always orthogonally tilted (150°) with respect to the grating at the attended location (60°). In the example shown here, the upper grating is part of the same perceptual object containing the attended grating (grouping condition), whereas the lower grating forms a distinct perceptual unit (no-grouping condition). (B) Schematic representation of the paradigm used in the test phase of Experiments 1 and 2. In each session, the three locations (attended, grouping, no-grouping) were tested separately, after the exposure phase was completed. At the location to be tested, two gratings appeared for 200 ms in a rapid sequence, separated by a 200-ms blank interval. The first grating had the same orientation presented, at the same spatial location, during the exposure phase. In half of the trials, the second grating was rotated with respect to the first grating and the orientation difference varied among the following values: ±5°, ±7°, ±9°, ±11°, ±13°. In the other half of the trials, the second grating was the same as the first one. Participants performed an orientation discrimination task (same vs. different).

Three sine-wave circular gratings (2 cpd; 3° in diameter) could appear at high (100%) or lower (80%) contrast, over a grey background (22 cd/m^2^). Stimuli were presented at 6.5° eccentricity on the left of the central fixation point (a small white disk of. 1° of visual angle). One grating was placed on the horizontal meridian, while the upper and lower gratings were located on two diagonal meridians, 45° above and 45° below the horizontal meridian, respectively (Fig. 1).

### Procedure

Each experiment included 5 sessions conducted on consecutive days. During each session, participants experienced an exposure (or training) phase and a test phase.

#### Exposure phase

In each session, the exposure phase consisted of 1500 trials divided into 5 consecutive blocks. In each block, which lasted 2 min, the stimuli were presented 300 times. For each presentation, the 3 equidistant gratings were displayed for 200 ms, followed by a 200-ms blank interval. Participants were asked to detect, as quickly as possible, the occurrence of lower-contrast gratings (the targets) at the location on the horizontal axis (the attended location) by pressing a key on the computer keyboard, while ignoring the upper and lower stimuli. Targets occurred independently at each location and were 10% of the total presentations (see also [33]). In Experiment 1, either the upper or lower grating (counterbalanced across participants) shared the same orientation as the attended grating (60°), whereas the remaining grating was orthogonally oriented (150°). In Experiment 2, both the upper and lower gratings were orthogonal (150°) to the attended grating (60°). However, the attended grating and either the upper or the lower grating (counterbalanced across participants) were surrounded by a black outline ellipse-like shape. The remaining grating was surrounded by a black outline circle (Fig. 1A).

#### Test phase

The test followed the exposure phase after a few minutes of rest. In each session, the test phase consisted of 900 trials divided into 3 consecutive blocks. On each trial within a block, two gratings lasting 200 ms and separated by a 200-ms delay were presented in rapid succession at one of the three locations occupied during the exposure phase. The first grating was always the one presented (for a given specific location) during the exposure phase. The second grating either was the same or was slightly tilted at ±5°, ±7°, ±9°, ±11°, ±13° with respect to the first grating. Participants performed an orientation discrimination task (same vs. different): they were instructed to indicate whether the second grating was tilted or not with respect to the first grating, by pressing two different buttons. Each location (‘attended’, ‘grouping’, ‘no-grouping’) was separately tested in different blocks, whose order was counterbalanced across participants. Each block consisted of 300 trials (50% “same” trials, and 50% “different” trials, with 30 trials for each of the 5 different orientations) (Fig. 1B).

### Attention criterion for the exposure phase

Following Gutnisky et al. [33], to ensure that during the exposure phase participants effectively attended to the designated grating, we computed the percentage of correct target detection (lower-contrast gratings) made by each participant. Participants were removed from the data set if, on average, they missed more than 20% of the targets over the five sessions. Four participants were removed from Experiment 1, and six were removed from Experiment 2. The averages for correct target detection were 92% and 91% in Experiments 1 and 2, respectively.

### Eye movements

Participants were instructed to maintain their eyes on the fixation point during both the exposure and test phase. Their fixation was monitored by means of an eye tracker, which detected any gaze deviation larger than 1.5°. If an eye movement towards the stimuli occurred, it was immediately signaled to the participant via an auditory feedback, and the corresponding trial was removed from the analysis. Overall eye movements were rare and this criterion removed less than 3% and 5% of the data in Experiments 1 and 2, respectively.

The eye tracker was re-calibrated at the end of each 2-min block during the exposure phase and after the test of each position in the test phase.

## Results

### Experiment 1

**Figure 2:**
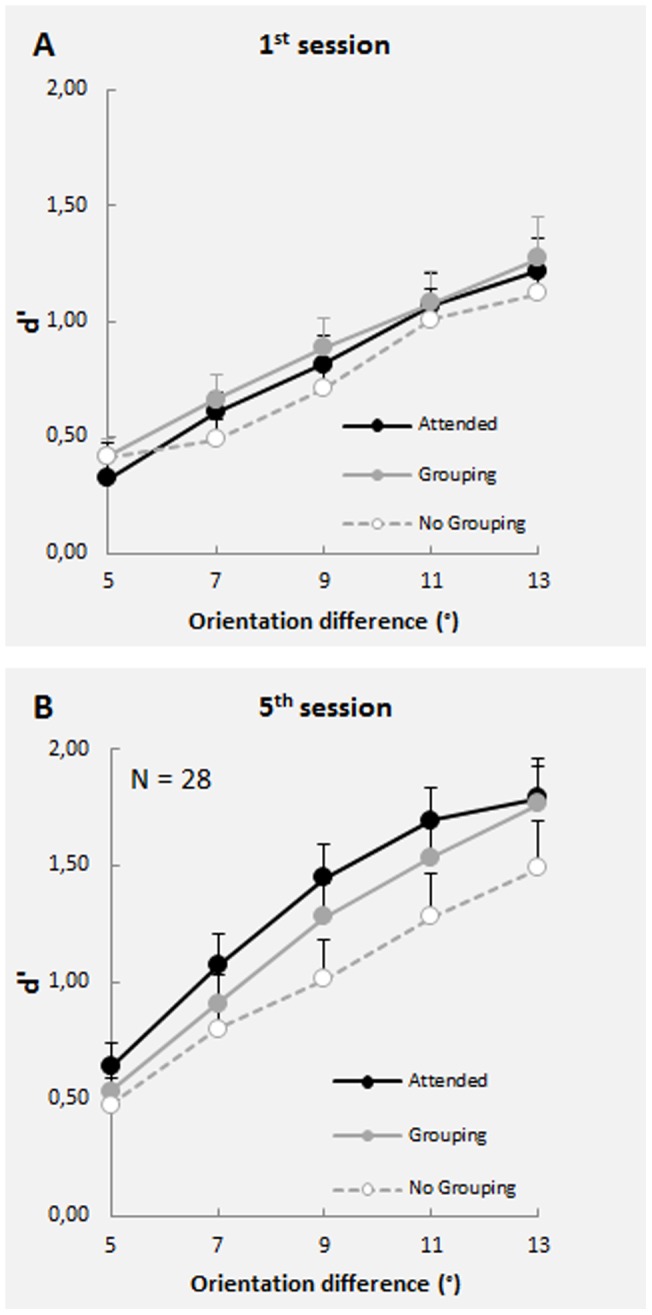
Results obtained from the first and last session of Experiment 1, when grouping was induced on the basis of orientation similarity. The mean orientation-discrimination performance (*d*') was plotted for each condition as a function of the orientation difference. Symmetric positive and negative orientation differences (e.g., ±5°) were pooled together. Error bars represent SEM. (A) In the 1^st^ session, the discriminative ability (*d*') of participants did not differ between conditions. (B) By the 5^th^ day of training, participants were better at discriminating orientation differences in the attended and grouping conditions than in the no-grouping condition.

In this experiment, perceptual grouping between the attended and unattended gratings was manipulated via orientation similarity. During the exposure phase, one of the two unattended gratings had the same orientation as the attended one (grouping condition), while the other grating had an orthogonal orientation (no-grouping condition). The results (Fig. 2A, B) showed that sensitivity (*d*') in orientation discrimination was modulated by attention [33] and more importantly, by perceptual grouping. A three-way ANOVA with Session, Condition, and Orientation difference as factors showed a significant interaction between Session and Condition, [*F*(8,216)  = 2.179, *P* = 0.03]. Performance did not differ between conditions in the 1^st^ session [*F*(2,54)  = 1.116, *P* = 0.335], but by the 5^th^ session participants' discriminative ability was better in the attended and grouping condition than in the unattended condition [*F*(2,54)  = 9.084, *P* = 0.001]. Pairwise comparisons (Bonferroni corrected) confirmed that in the last session performance was higher, compared to the no-grouping condition, in both the attended (*P* = 0.001) and grouping condition (*P* = 0.038). These results support the hypothesis that perceptual grouping can modulate perceptual learning, inducing a higher degree of plasticity for an unattended stimulus perceptually grouped with the attended one.

However, before accepting this conclusion an alternative explanation must be excluded. While attentional deployment can explain the difference in performance between the attended and the no-grouping condition [33], the significant improvement observed in the grouping condition could be explained by learning transfer from the attended location. Indeed, although perceptual learning is usually specific for retinal position [20], some degree of transfer may occur in areas near the trained location [34]. Additionally, because the grating tested in the grouping condition was the same as the grating attended during the training session, the transfer hypothesis could account for the results of Experiment 1 without invoking any modulatory role for perceptual grouping on perceptual learning.

### Experiment 2

**Figure 3:**
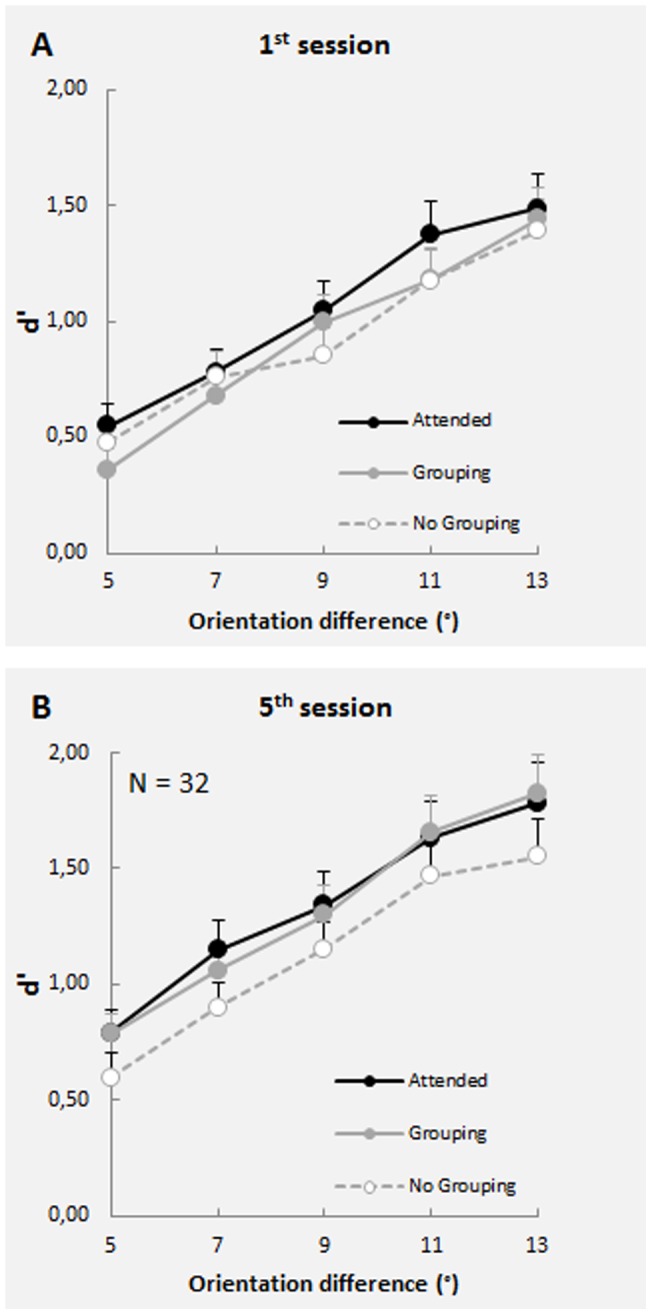
Results obtained from the first and last session of Experiment 2, when grouping was induced by a surrounding frame. The curves represent the mean discriminative ability (*d*') measured in the three different conditions. The data coming from symmetric orientation differences (e.g., ±5°) were pooled together. Error bars represent SEM. (A) No differences between conditions emerged in the 1^st^ session. (B) By the 5^th^ session, performance increased in the attended and grouping conditions when compared to the no-grouping condition.

To exclude learning transfer as an explanation of our results, we conducted a second experiment in which both unattended gratings had the same orientation, orthogonal to that of the attended one. In this experiment, perceptual grouping was obtained by surrounding one of the two unattended gratings and the attended one with the outline of an ellipse-like shape, making them part of the same perceptual object (Fig. 1A). Our results (Fig. 3A, B) replicated those of the previous experiment, showing the effect of attention and perceptual grouping. A three-way ANOVA showed a significant interaction between Session and Condition [*F*(8,248)  = 2.034, *P* = 0.043]. Conditions were not statistically different in the 1^st^ session [*F*(2,62)  = 1.387, *P* = 0.258], but they were different in the 5^th^ session [*F*(2,62)  = 4.672, *P* = 0.013]. Pairwise comparisons showed that the attended and grouping conditions did not differ from each other, while sensitivity in both conditions was higher than in the no-grouping condition (*P* = 0.011 and *P* = 0.052, respectively. Bonferroni corrected). To substantiate the marginally significant difference between grouping and no-grouping conditions, we also performed an alternative statistical analysis based on a Bayesian method [35]. We calculated the posterior probability that the two conditions do not differ from each other (null hypothesis H_0_), given the collected data set (D), and the complementary posterior probability that the two conditions do differ (alternative hypothesis H_1_). We obtained the following values: p(H_0_|D)  = 0.22 and p(H_1_|D)  = 1 – p(H_0_|D)  = 0.78. These posterior probability values indicate that our data set clearly favor the alternative hypothesis over the null hypothesis, thus corroborating the results emerged from the (extremely conservative) Bonferroni-correction.

## Discussion

Previous studies on visual perceptual learning have identified attention and reinforcement signals as two factors controlling the improvement in discriminative performance due to training [12]. Here, we showed that perceptual grouping is another important factor that can modulate the learning process. In particular, our data indicated that perceptual grouping induced transfer of learning to an untrained spatial position.

Perceptual learning is often specific for the actual parameters (e.g., position, orientation) of the trained stimulus (but see [36]). However, a certain degree of transfer can be observed either as a function of the presentation regime [37], [38], or because of a double-training procedure [39]. In the present study we found that perceptual grouping can favor the transfer of learning from the attended location to an untrained location that was perceptually grouped with the attended one. One possible interpretation is that perceptual grouping can directly modulate, at pre-attentive stages of visual processing, perceptual learning. In line with this hypothesis, there is converging behavioral evidence showing that visual grouping can occur without attention [40], [41]. In our paradigm, learning may have pre-attentively been transferred from the attended stimulus to the unattended-grouped stimulus, simply by virtue of the displayed perceptual organization.

Alternatively, it is possible that the effect shown by our data was mediated by attention. Two lines of evidence support this possibility. First, it is well established that the initial grouping process that results in perceptual organization can constrain the deployment of attention [31], [42]–[44]. Attention automatically spreads towards perceptually grouped visual units or to distant parts of the same attended object [32], and this may have occurred in the displays used in our experiments. Second, single-cell recording studies have shown that attention and perceptual-grouping factors interact in early visual areas [45], [46]. In particular, in agreement with the findings of previous behavioral studies [31], [42]–[44], a recent neurophysiological study on the activity of primary visual cortex (V1) in the macaque monkey, found evidence of an automatic spread of attention to stimuli that were perceptually grouped with the task-relevant item [46].

Our findings cannot distinguish between the pre-attentive and the attentive explanation. However, one may note that in object-based attention experiments the effects of attention are usually smaller in the grouping condition than in the position directly attended (e.g., [47]). By contrast, in our experiments the amount of learning in the attended and grouping conditions was almost equivalent, which might suggest that in our study learning was directly affected by grouping. Even if one were to accept that in our experiments the effect of grouping was mediated by attention, it is important to note that the type of attention involved in our study appears to be different from the one previously proposed for the control of perceptual learning. In contrast with previous studies indicating that a voluntary effort to direct focused attention to the relevant feature is crucial for perceptual learning [13]–[15], our data showed that such voluntary control of attention is not always necessary. In our paradigm, any shift of attention from the task-relevant stimulus to the grouping stimulus must have occurred automatically [31] rather than through voluntary effort, given that participants were explicitly instructed to ignore task-irrelevant stimuli. Nonetheless, perceptual learning was larger in the grouping condition than in the no-grouping condition, and its effect was comparable to that observed for the task-relevant stimulus that was intentionally attended.

In conclusion, this study showed that perceptual grouping, either directly or via automatic attention, is another important factor that controls perceptual learning.
